# The Effect of Flap Elevation on the Ocular Cyclotorsion in Customized Laser Ablation

**DOI:** 10.3390/jcm14082596

**Published:** 2025-04-10

**Authors:** Noa Kapelushnik, Dana Barequet, Ami Hirsh, Israel Kremer, Ori Mahler, Samuel Levinger, Irina S. Barequet

**Affiliations:** 1 Goldschleger Eye Institute, Sheba Medical Center, Tel Hashomer 52621, Israel; kapelushniknoa@gmail.com; 2 Faculty of Medical and Health Sciences, Tel Aviv University, Tel Aviv 69978, Israel; danabarequet@gmail.com; 3 Division of Ophthalmology, Tel Aviv Sourasky Medical Center, Tel Aviv 6423906, Israel; 4 Enaim Refractive Surgery Center, Jerusalem 9438307, Israel; amihirsh@gmail.com (A.H.); mk@barak.net.il (I.K.); dr.mahler@cataract.org.il (O.M.); callcenter@enaim.co.il (S.L.)

**Keywords:** refractive surgery, LASIK, PRK, cyclotorsion, ophthalmology

## Abstract

**Objectives**: Corneal refractive surgery aims to correct refractive errors. Proper corneal alignment is crucial. Eye-tracking technologies, specifically designed to address cyclotorsion using iris registration, help reduce the effects of cyclotorsion during surgery. The timing of iris registration can influence the efficacy of these technologies. This study compared cyclotorsion measurements before and after flap elevation/epithelium removal in FemtoLASIK and alcohol-assisted (aa) PRK. **Methods**: This retrospective cohort study was conducted at Einaim Medical Centers, Israel, and included patients who underwent refractive surgery using the VISX Star S4 IR excimer laser. Cyclotorsion measurements were obtained pre- and post-flap elevation or removal using the Wavescan™ and VISX Star S4 iris registration systems. Patients’ data were collected and analyzed retrospectively. **Results**: Overall, 152 eyes of 86 patients were included. In the FemtoLASIK group, 73 eyes from 45 patients were analyzed. For pre-flap lift, 34.2% had incyclotorsion and 65.8% had excyclotorsion, with a mean cyclotorsion of 2.3 ± 1.5 degrees. For post-flap lift, the mean cyclotorsion was 2.8 ± 1.9 degrees, showing a significant difference (*p* = 0.01). In the aa-PRK group, 79 eyes from 41 patients were analyzed. For pre-flap removal, 45.6% had incyclotorsion and 53.2% had excyclotorsion, with a mean cyclotorsion of 2.6 ± 1.8 degrees. For post-flap removal, the mean cyclotorsion was 2.5 ± 2.1 degrees, with no significant difference (*p* = 0.47) and a mean change of 1.6 ± 1.2 degrees. A total of 15.2% of eyes in the aa-PRK group and 13.6% in the LASIK group exhibited more than 3 degrees of cyclotorsional difference before and after flap lift or epithelial removal. **Conclusions**: Cyclotorsion occurs after flap lift/removal. To minimize residual astigmatism, iris registration should be performed post-flap elevation.

## 1. Introduction

In recent years, the goals of corneal refractive surgery have expanded beyond merely correcting simple refractive errors like myopia, hyperopia, and astigmatism. The focus has shifted toward achieving optimal or near-optimal vision quality and addressing higher-order aberrations, a development made possible by advancements in excimer laser technology [1]. The commonly performed kerato-refractive surgeries, femtosecond laser-assisted in situ keratomileusis (FemtoLASIK), and Photorefractive Keratectomy (PRK) allow treatments to be tailored to individual corneal properties, enabling the correction of high astigmatism and higher-order aberrations [2]. LASIK emerged as a groundbreaking refractive surgical technique in the 1990s, integrating the biomechanical principles of radial keratotomy with the precision and predictability of excimer laser technology. This innovation enabled controlled corneal reshaping to correct refractive errors with enhanced accuracy. By the late 1990s, LASIK had become widely adopted as a minimally invasive procedure capable of effectively addressing myopia, hyperopia, and astigmatism. Its rapid acceptance was driven by its ability to provide rapid visual recovery, reduced postoperative discomfort, and improved predictability compared to earlier refractive techniques. The continuous advancements in laser technology, including femtosecond laser flap creation and wavefront-guided ablation profiles, have further refined the procedure, improving both safety and refractive outcomes [3].

Photorefractive Keratectomy (PRK) is a well-established corneal refractive procedure that serves as an alternative to laser-assisted in situ keratomileusis (LASIK), particularly for patients with thinner corneas or specific lifestyle requirements. Unlike LASIK, which involves the creation of a corneal flap, PRK entails the removal of the corneal epithelium to access the underlying stromal tissue. The excimer laser is then used to precisely reshape the cornea based on the patient’s refractive error [4].

LASEK (Laser-Assisted Sub-Epithelial Keratectomy) is another common refractive surgery that combines elements of both PRK and LASIK [5]. Like PRK, LASEK avoids the creation of a stromal flap, making it suitable for patients with thin corneas or those with other contraindications for LASIK surgery. However, unlike PRK, where the corneal epithelium is completely removed and discarded, LASEK, in most of the techniques, involves the application of an alcohol solution to loosen the epithelial layer, which is then gently lifted and preserved [6,7]. After laser ablation, the epithelium is repositioned over the treated area [8].

Proper corneal alignment during all refractive surgeries is essential, particularly when addressing high astigmatism and aberrations. However, this can be challenging due to involuntary eye movements, particularly cyclotorsion. These movements are exacerbated by the transition from preoperative measurements, conducted in an upright position, to surgery performed in a supine position, as well as the monocular viewing conditions during surgery [1,2]. To maintain measurement accuracy despite these positional changes, eye-tracking technologies, especially those utilizing iris registration modalities, are widely used [9]. Eye-tracking technology has become a fundamental component of refractive surgery, substantially improving the precision and efficacy of surgical outcomes. These systems employ sophisticated algorithms and high-speed hardware to continuously monitor ocular movements in real-time, enabling surgeons to dynamically compensate for involuntary eye motions, including saccadic shifts and changes in pupil position. By integrating real-time adjustments, eye-tracking enhances treatment accuracy, ensuring optimal alignment and delivery of the laser ablation profile tailored to each patient’s unique ocular dynamics [10]. Cyclotorsion of the eye can occur during head and body movements, with reported average rates of approximately 15 degrees, though this varies among individuals. The refractive surgery process itself may also induce cyclotorsion, potentially causing residual astigmatism if left unaddressed [11]. It is standard practice to perform iris registration after flap elevation and immediately before the laser ablation to ensure the most accurate alignment [12]. However, cyclotorsion measurements may be influenced by the flap creation process, and performing registration after flap elevation poses the additional risk of corneal stroma dehydration. This study aims to evaluate the differences in cyclotorsion measurements before and after flap elevation or removal in two types of refractive surgeries: FemtoLASIK and alcohol-assisted (aa) PRK.

## 2. Materials and Methods

This retrospective cohort study included consecutive patients who underwent refractive surgery for the correction of myopia or hyperopia, with or without astigmatism. All procedures were performed using the VISX Star S4 IR excimer laser system (Abbott Medical Optics Inc., Santa Ana, CA, USA), which incorporates advanced wavefront-guided ablation and eye-tracking technology to optimize treatment precision. The study received approval from Sheba Medical Centre’s institutional review board and was conducted in accordance with the tenets of the Declaration of Helsinki. Due to the retrospective nature of the study, the requirement for informed consent was waived.

### 2.1. Data Collection

Patient data were reviewed for all eyes that underwent either alcohol-assisted PRK (aa-PRK) or FemtoLASIK during the study period. Inclusion criteria were patients aged 18 years or older, with a minimum of two months of postoperative follow-up, and availability of cyclotorsion measurements before and after flap elevation/removal. Data collected included baseline demographics, preoperative refractive error, cyclotorsion measurements at different surgical stages, and postoperative visual acuity outcomes.

### 2.2. Surgical Technique

In both procedures, the patient provided written informed consent by signing a detailed operation form. All surgeries were conducted by the same 4 experienced surgeons at the same surgical center during the study period.

#### 2.2.1. FemtoLASIK

Under topical anesthesia (Localin; Oxybuprocaine hydrochloride 0.4%; Fischer Labs Ltd., Tel Aviv, Israel), a corneal flap was created using the IntraLase femtosecond laser system (IntraLase Corp., Irvine, CA, USA). The laser operated at a 15 kHz repetition rate in a raster pattern, with a pulse separation of 10 × 10 µm, side-cut energy of 3.6 µJ, and bed energy of 2.5 µJ. The flap design included a 45° superior hinge angle, 70° side-cut angle, and a 9 mm diameter to ensure adequate exposure of the stromal bed. Iris registration, an integral feature of the VISX platform, was performed under mesopic conditions both before and after flap elevation to account for any cyclotorsion misalignment. Excimer laser ablation was subsequently performed using the wavefront-guided VISX Star S4 CustomVue platform, which incorporates active eye-tracking to maintain laser accuracy during treatment. Postoperative regimen included 0.3% ofloxacin and 0.1% dexamethasone eye drops, four times daily for seven days, and non-preserved artificial tears for symptomatic relief and corneal hydration.

#### 2.2.2. Alcohol-Assisted PRK (aa-PRK)

Iris registration was performed prior to ethanol application and epithelial removal to ensure proper alignment. Under topical anesthesia, a 20% ethanol solution was applied using an 8.5 mm fixation well for 35 s, followed by irrigation with balanced salt solution (BSS). The corneal epithelium was then mechanically removed using a blunt spatula, followed by a repeat iris registration before excimer laser ablation. Laser ablation was conducted using the wavefront-guided VISX Star S4 CustomVue platform, with real-time eye-tracking adjustments to compensate for micro-movements during treatment. To mitigate the risk of postoperative corneal haze, 0.02% Mitomycin-C (MMC) was applied to the stromal bed for 10 to 30 s, depending on the depth of stromal ablation. The stromal bed was then thoroughly irrigated with 15 cc of chilled BSS. A bandage contact lens (PureVision, Bausch & Lomb, Inc., Rochester, NY, USA) was placed on the corneal surface following instillation of 0.1% dexamethasone sodium phosphate and 0.5% moxifloxacin hydrochloride eye drops. Postoperative regimen included 0.3% ofloxacin and 0.1% dexamethasone eye drops, four times daily for seven days, and non-preserved artificial tears to promote re-epithelialization and comfort.

### 2.3. Cyclotorsion Measurements

All patients underwent a comprehensive preoperative ophthalmic examination, including wavefront aberration analysis and infrared iris imaging using the Wavescan™ system (Abbott Medical Optics Inc., Santa Ana, CA, USA) while seated.

During surgery, iris registration was conducted with the VISX Star S4 system, which compensates for static and dynamic cyclotorsion when transitioning from an upright to a supine position. Preoperative iris images served as reference points, while intraoperative images captured after flap elevation (FemtoLASIK) or epithelial removal (aa-PRK) were used for static cyclotorsion measurements.

The VISX Star S4 system employs an advanced algorithm to determine the torsional angle and adjust laser pulse coordinates accordingly to maintain alignment with the intended ablation profile [13]. Cyclotorsion changes were calculated as the difference between pre- and post-flap elevation/removal measurements, ensuring precise ablation placement.

### 2.4. Statistical Analysis

Data analysis was performed using SPSS version 25.0 (SPSS, Inc., Chicago, IL, USA). Paired *t*-tests were used to assess the differences in cyclotorsion measurements before and after flap elevation/removal within each surgical group. Independent sample *t*-tests were used to compare the absolute cyclotorsion differences between the FemtoLASIK and aa-PRK groups. Pearson’s correlation coefficient was employed to evaluate potential confounders for continuous variables, while univariate logistic regression was used for categorical variables. All data are presented as mean ± standard deviation (SD), and statistical significance was defined as *p* < 0.05.

## 3. Results

### 3.1. Demographics

A total of 152 eyes were included in the study, with 79 eyes undergoing aa-PRK and 73 eyes undergoing FemtoLASIK. The mean age of patients was 28.8 ± 9.7 years, with those in the aa-PRK group being significantly younger (25.2 ± 5.8 years) compared to the FemtoLASIK group (32.8 ± 11.6 years). The distribution of sexes was equal across the entire cohort (50% male, 50% female) and similar between groups (51.2% male in aa-PRK vs. 48.9% male in FemtoLASIK). Table 1 describes the demographic and surgical data of the included eyes in the study.

### 3.2. FemtoLASIK

**Figure 1:**
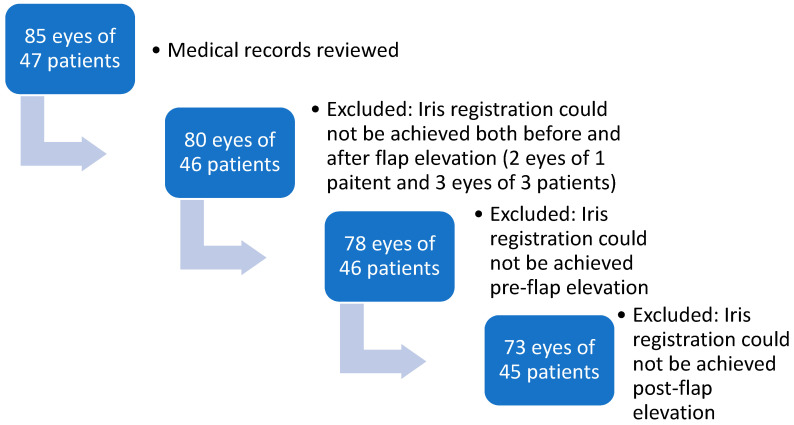
DireFlowchart of FemtoLASIK study population.

**Figure 2:**
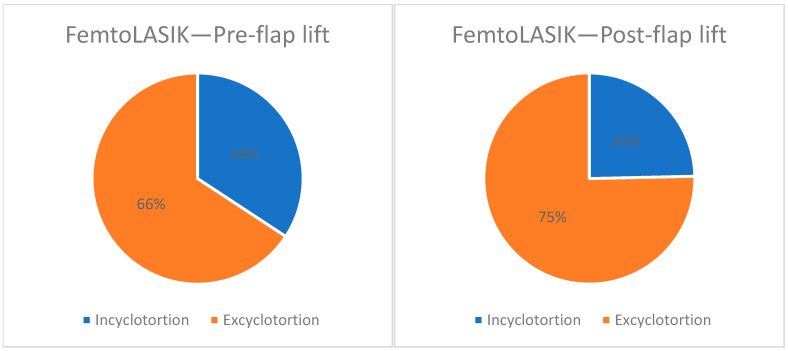
Direction of cyclotorsion before and after flap lift in FemtoLASIK.

Medical records of 87 consecutive surgeries of 47 patients were reviewed. Among these, 73 eyes of 45 patients were included in the study (excluded eyes are explained in Figure 1). Twenty-one patients (46%) were male. Mean age at surgery was 32.8 ± 11.7 years (range, 18 to 56 years). The preoperative sphere ranged from −9.5 to +5 D. Eighty five percent (85%) of the patients were myopic and the mean sphere among the myopic patients was −3.7 ± 2.8 D (range, −9.5 to −0.25 D) and the mean cylinder was −1.6 ± 1.1 D (range −5 to −0.25 D). The mean sphere among the hyperopic patient was +2.6 ± 1.2 D (range, +1.25 to +5 D) and the mean cylinder was −0.8 ± 0.7 D (range −2.5 to −0.25 D). Before flap lift, 25 eyes (34.2%) had incyclotorsion and 48 (65.8%) had excyclotorsion. The mean cyclotorsion measured was 2.3 ± 1.5 degrees (range’ 0 to 5.9 degrees). After flap elevation, 7 eyes changed from incyclotorsion to excyclotorsion, changing the trend to 75.3% of the eyes which had excyclotorsion (Figure 2). The other kept the direction of the cyclotorsion measured before flap elevation. The mean cyclotorsion measured after flap elevation was 2.8 ± 1.9 degrees (range, 0 to 7 degrees). The difference between the measurements before flap elevation and after flap elevation is statistically significant (*p* = 0.01). The mean difference is 1.6 ± 1.4 degrees (range 0 to 7.1 degrees). In 13.6% of the eyes, a cyclotorsion difference of more than 3 degrees was noted.

The difference in cyclotorsion before flap lift and after flap lift was not significantly correlated with the patient’s age or gender but there is a weak negative correlation between the preoperative sphere and the difference (r = −0.276, *p* value = 0.023, Pearson correlation).

### 3.3. aa-PRK

**Figure 3:**
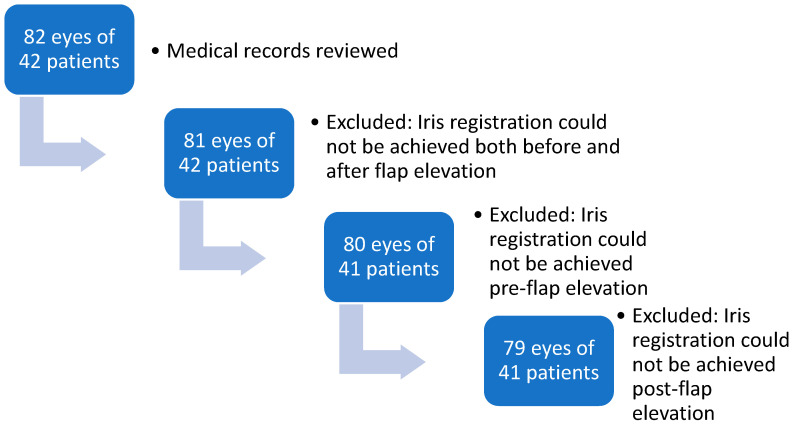
Flowchart of aa-PRK study population.

**Figure 4:**
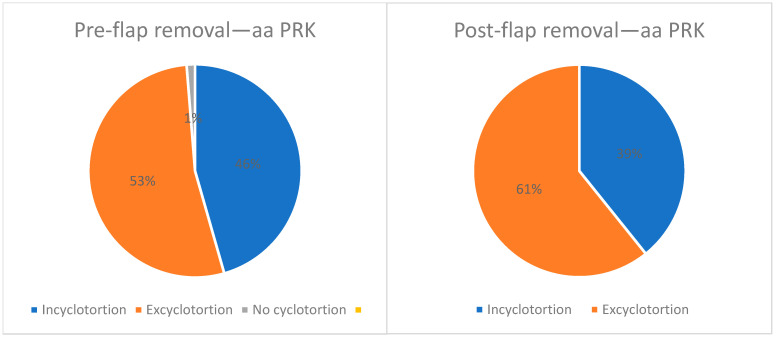
Direction of cyclotorsion before and after flap removal in aa PRK.

Medical records of 82 consecutive surgeries involving 42 patients were reviewed. Of these, 79 eyes from 41 patients were included, with the exclusions detailed provided in Figure 3. Twenty-one (51.2%) were male. The mean age at surgery was 25.2 ± 5.8 years. The preoperative sphere ranged from −7.5 to +2.25 D. Among the participants, 97.5% had a mean sphere of −3.2 ± 1.6 D (range’ −7.5 to −0.75 D) and the mean cylinder was −0.8 ± 0.7 D (range −0.5 to −3.25 D). Only one eye was hyperopic with +2.25 D, and the cylinder was −3.7 D. Before flap removal, 36 eyes (45.6%) had incyclotorsion and 42 (53.2%) had excyclotorsion. One eye (1.3%) had no cyclotorsion (0 degrees). The mean cyclotorsion measured was 2.6 ± 1.8 degrees (range, 0 to 7.7 degrees). After flap removal, eight eyes changed from incyclotorsion to excyclotorsion, and two eyes changed from excyclotorsion to incyclotorsion (Figure 4). After the epithelial flap removal, 31 eyes (39.2%) had incyclotorsion and 48 (60.8%) had excyclotorsion. The mean cyclotorsion measured after flap removal was 2.5 ± 2.1 degrees (range, 0.1 to 8.6 degrees). The difference between the measurements before flap removal and after flap removal is not statistically significant (*p* = 0.47). The mean difference is 1.6 ± 1.2 degrees (range, 0 to 6 degrees). Cyclotorsion differences exceeding 3 degrees were observed in 15.2% of eyes.

The difference in cyclotorsion before flap removal and after flap removal is not significantly correlated with the patient’s age or gender but there is a weak negative correlation between the preoperative sphere and the difference (r = −0.233, *p* value = 0.04, Pearson correlation).

### 3.4. aa-PRK vs. FemtoLASIK

**Figure 5:**
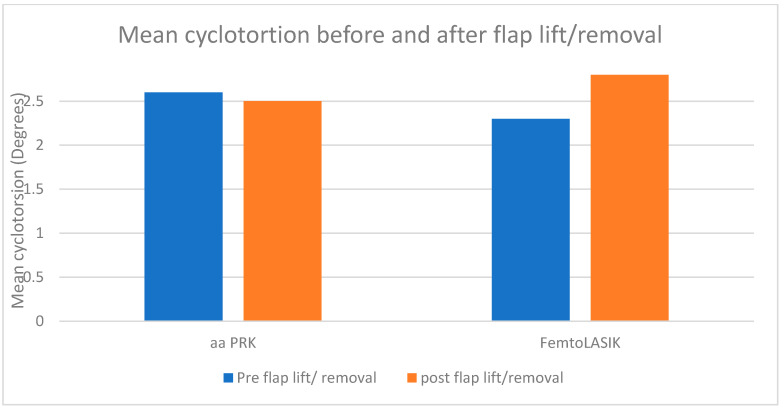
Mean cyclotorsion before and after flap lift/removal in aa PRK and FemtoLASIK surgeries.

There were no significant differences in cyclotorsion measurements before flap elevation and after flap elevation/epithelium removal between the aa-PRK operated eyes and FemtoLASIK eyes (*p* = 0.65, Levene’s Test for Equality of Variances). Figure 5 graphically describes cyclotorsion measured before and after flap lift/removal.

## 4. Discussion

This study aimed to evaluate the changes in cyclotorsion measurements before and after flap elevation/removal in two types of refractive surgeries, FemtoLASIK and aa-PRK, with a focus on understanding the importance of the timing for iris registration.

The findings revealed significant differences in cyclotorsion measurements pre- and post-flap elevation in the FemtoLASIK group. Specifically, the mean cyclotorsion difference was 1.6 ± 1.4 degrees. Conversely, the aa-PRK group did not show a statistically significant difference in cyclotorsion measurements before and after the epithelial flap removal with a difference in cyclotorsion of 1.6 ± 1.2 degrees. The difference in the statistical significance might be attributed to the manipulation in flap lift during FemtoLASIK, which might take longer than in aa-PRK, allowing more cyclotorsion to accrue. The weak negative correlation between the preoperative sphere and cyclotorsion differences in both surgical groups suggests that higher myopia may slightly reduce the variability in cyclotorsion changes. However, this correlation was not strong enough to be clinically significant.

In both groups, the direction of cyclotorsion was most dominantly excyclotorsion. Chernyak et al. used Visx Star S3 to evaluate the amount of cyclotorsion occurring between the wavefront measurements and the laser refractive surgery [14]. Their results also showed a trend towards excyclotorsion, with approximately 79% of eyes having excyclotorsion.

Misalignment of 3 degrees of the ablation may cause almost 10% of astigmatism under correction [15,16]. Such under correction has high potential of being clinically significant and affecting the visual outcome of the refractive procedure. Although the difference in cyclotorsion was not significant in the aa-PRK group, 15.2% had more than 3 degrees difference in cyclotorsion measurements before and after epithelial flap removal, meaning that despite the mean change in cyclotorsion measurements before and after epithelial flap removal, in a relatively large number of eyes, the change was clinically significant. In the FemtoLASIK group, 13.6% of the eyes had more than 3 degrees difference, which is also clinically significant. These results suggest that the clinical influence is important, and the amount of cyclotorsion has clinical importance that should be addressed during refractive surgery, using iris registration.

According to several studies, iris registration during refractive surgery has a positive impact on the visual outcome and postoperative high order aberrations [17,18].

Iris registration technology enhances the precision of refractive surgeries by compensating for ocular cyclotorsion and pupil center shifts that occur between preoperative measurements and the surgical procedure. By aligning the laser treatment to stable ocular landmarks, such as the iris, this method ensures accurate ablation positioning, leading to improved visual outcomes. Studies have demonstrated that wavefront-guided LASIK with iris registration induces fewer higher-order aberrations compared to conventional LASIK and femtosecond-assisted LASIK procedures [19]. Furthermore, utilizing multiple ocular landmarks for alignment, as facilitated by iris registration, significantly reduces alignment errors caused by pupil center movement and cyclotorsion, thereby increasing the safety and effectiveness of wavefront-based laser vision correction [20].

Khalifa et al. examined the efficiency of wavefront-guided laser in situ keratomileusis (LASIK) with iris registration technology to correct mixed astigmatism. They found that patients operated with the use of iris registration technology had better postoperative visual acuity. Brunson et al. demonstrated that the use of the VARIO imaging system for iris registration significantly reduced variability in clinical outcomes following wavefront-optimized LASIK. The system compensates for ocular cyclotorsion by capturing detailed iris landmarks preoperatively and matching them intraoperatively, thereby ensuring accurate alignment of the ablation profile. In their study, eyes treated with iris registration showed a lower incidence of residual refractive cylinder greater than 0.50 D and a higher proportion of eyes achieving better uncorrected and best-corrected visual acuity. These outcomes suggest that integrating iris registration technology—particularly with advanced imaging systems like VARIO—may enhance refractive precision and visual results by addressing both cyclotorsional shifts and centration accuracy during surgery [17]. Arba-Mosquera et al. also concluded that iris registration using SCHWIND AMARIS is beneficial and visual results are better [21]. The SCHWIND AMARIS laser system incorporates a sophisticated eye registration and tracking module designed to measure and compensate for both static and dynamic cyclotorsional movements during laser refractive surgery. Static cyclotorsion, which occurs when a patient moves from an upright to a supine (surgical) position, is measured by comparing a diagnostic image with an intraoperative image obtained just before ablation begins. Multiple measurements are taken to ensure accuracy, and the system applies strict criteria for accepting values into treatment planning, particularly when cyclotorsion exceeds 5 degrees or when repeatability is poor. Dynamic cyclotorsion, which refers to rotational eye movements during the ablation itself, is tracked continuously by comparing real-time images to the initial baseline at the start of treatment. The system registers and compensates for these movements with high sensitivity.

This dual compensation approach not only minimizes the mismatch between planned and delivered ablation profiles but also reduces rotational misalignments. Arba-Mosquera et al. further observed that dynamic cyclotorsion often partially counterbalanced static cyclotorsion, highlighting the importance of tracking both components rather than relying solely on static measurements.

Although many studies have demonstrated the clinical benefit of iris registration and cyclotorsion compensation in enhancing the accuracy of refractive corrections, particularly in astigmatic treatments, the findings of Lee et al. [12] suggest a different outcome. In their study evaluating wavefront-guided LASEK using the VISX Star S4 system, they found no significant difference in key refractive outcomes such as uncorrected visual acuity, residual cylinder, or higher-order aberrations, between eyes treated with and without IR. The mean cyclotorsion observed in their IR group was relatively low (2.1°), and the authors speculated that under such well-controlled surgical conditions, static cyclotorsional compensation may not confer a measurable clinical advantage. These findings imply that the utility of IR may depend on factors such as the degree of cyclotorsion, the surgical technique, and the refractive profile of the patient, and may be more relevant in cases involving greater rotational shifts or higher optical complexity.

In this study, we compared the amount of cyclotorsion in aa-PRK procedure and FemtoLASIK before and after flap lift or removal. Narváez et al. measured the amount of cyclotorsion before and after flap creation in LASIK procedure and after epithelial removal in PRK procedure [22]. While both studies evaluated cyclotorsion during the LASIK procedure, there are important methodological differences. Narváez et al. measured cyclotorsion before and after flap creation, whereas in the present study, measurements were taken before and after flap elevation. Additionally, a key distinction lies in the technique used for the flap. Narváez et al. utilized the MK2000 mechanical microkeratome, whereas in our study, the flap was created using the IntraLase femtosecond laser system (IntraLase Corp., Irvine, California; NIDEK Inc., Fremont, California). As surgical technologies evolve, so too do the techniques employed in refractive surgery. By using a more advanced and currently widespread method for flap creation, our study provides updated insights into cyclotorsional changes during modern FemtoLASIK procedures. Moreover, the sample size in our study was notably larger, with 73 eyes in the FemtoLASIK group compared to 28 eyes in the LASIK group in the previous study. Similarly, our PRK group included 79 eyes, whereas Narváez et al. evaluated 31 eyes in their PRK group. In their study, as in ours, no significant difference in the amount of cyclotorsion was observed when comparing LASIK and PRK. Both studies reported a significant cyclotorsional change following flap elevation in LASIK. However, unlike our findings, Narváez et al. also identified a significant cyclotorsional shift in PRK after epithelial removal. This discrepancy may reflect differences in methodology, patient populations, or sample size.

Hyojin et al. evaluated the amount of cyclotorsion before and after flap creation during LASIK surgery in a similar method as in this study [11]. They found that the amount and direction of cyclotorsion is influenced after flap lift as in this study. They concluded that it is better to assess iris registration after flap lift because of the cyclotorsion change. In contrast to our study, which compared cyclotorsional changes in both FemtoLASIK and PRK procedures, the study by Hyojin et al. focused solely on LASIK and did not include a PRK comparison. Furthermore, while our study utilized a femtosecond laser (IntraLase) for flap creation, a method that reflects current standard practice, Hyojin et al. employed a mechanical microkeratome (M2, Moria) to create a 130-micron thick flap. This distinction in flap creation technique may influence the degree of cyclotorsional movement observed and highlights the relevance of our findings in the context of modern surgical technology.

Rotational movement of the eye may occur during the refractive ablation itself, potentially causing misalignment and residual astigmatism. Dynamic intraoperative iris registration can offer an option to compensate for intraoperative changes in cyclotorsion [23]. Obtaining iris registration is usually easier prior to flap lift, since after the flap lift, the corneal stroma is exposed and can be dehydrated, making iris registration more challenging. It is reasonable to assume that iris registration becomes more challenging in PRK following epithelial removal due to corneal haze or clouding. This is supported by the findings of Narváez et al. [22], who reported successful iris registration in 100% of eyes prior to epithelial removal, but in only 93.9% of eyes after the epithelium was removed. Fahd et al. assessed in their study the amount of “no catch” in iris registration before and after flap lift in LASIK cases. They found that the rates of iris registration failure after flap lift was higher than before flap lift [24]. Although obtaining iris registration as close as possible to the laser treatment can produce more accurate results and less residual astigmatism due to misalignment, the time of exposed stroma can reduce the chance of successful registration.

Our study has some limitations. Its retrospective nature is one limitation. Additionally, all patients underwent the surgery according to iris registration post-flap lift/removal, and there was no comparison between the clinical outcomes of iris registration obtained before and after the flap lift. Future studies should be prospective and compare the visual outcomes of obtaining iris registration before and after flap lift, including not only visual acuity but also higher-order aberrations, which can be influenced by misalignment caused by cyclotorsion

## 5. Conclusions

In conclusion, our study demonstrated that clinically significant cyclotorsion occurs after flap lift/removal both in FemtoLASIK and aa-PRK. Since this cyclotorsion has the potential to cause residual astigmatism and despite the risk of longer stromal exposure, it is advised to obtain iris registration as close as possible to the laser treatment—after flap elevation.

## Figures and Tables

**Table 1:** Surgical and demographic data of eyes included in study.

			aa-PRK	FemtoLASIK	All
Demographics	Number of eyes		79	73	152
	Age (Mean ± SD)		25.2 ± 5.8	32.8 ± 11.6	28.8 ± 9.7
	Sex	Male n (%)	21 (51.2%)	22 (48.9%)	43 (50%)
		Female n (%)	20 (48.7%)	23(51.1%)	43 (50%)
Preoperative	Sphere (D)		−3.1 ± 1.7	−2.7 ± 3.5	−2.9 ± 2.6
	Cylinder (D)		−0.9 ± 0.8	−1.5 ± 1.1	−1.2 ± 1
Postoperative	VA (LogMar)		0.01 ± 0.05	0.03 ± 0.07	0.02 ± 0.06
Cyclotorsion measurements	Pre-flap lift	Incyclotorsion n (%)	36 (45.6%)	25 (34.2%)	61 (40.1%)
		Excyclotorsion n (%)	42 (53.2%)	48 (65.8%)	90 (59.2%)
		Degrees (Mean ± SD)	2.6 ± 1.8	2.3 ± 1.5	2.5 ± 1.7
	Post-flap lift	Incyclotorsion n (%)	31 (39.2%)	18 (24.6%)	49 (32.2%)
		Excyclotorsion n (%)	48 (60.8)	55 (75.3%)	103 (67.8%)
		Degrees (Mean ± SD)	2.5 ± 2.1	2.8 ± 1.9	2.6 ± 1.9
	Change in cyclotorsion (|pre-flap lift-post-flap lift, degrees|)		1.6 ± 1.2	1.6 ± 1.4	1.6 ± 1.3

## Data Availability

Data are available upon reasonable request from the author.
